# Inverted (Reverse) Takotsubo Cardiomyopathy following Cerebellar Hemorrhage

**DOI:** 10.1155/2014/781926

**Published:** 2014-03-11

**Authors:** Sophie Piérard, Marco Vinetti, Philippe Hantson

**Affiliations:** ^1^Department of Intensive Care, Université Catholique de Louvain, Cliniques Universitaires Saint-Luc, Avenue Hippocrate 10, 1200 Brussels, Belgium; ^2^Louvain Centre for Toxicology and Applied Pharmacology, Université Catholique de Louvain, Cliniques Universitaires Saint-Luc, 1200 Brussels, Belgium

## Abstract

*Background*. First described in 2005, inverted takotsubo is one of the four stress-induced cardiomyopathy patterns. It is rarely associated with subarachnoid hemorrhage but was not previously reported after intraparenchymal bleeding. *Purpose*. We reported a symptomatic case of inverted takotsubo pattern following a cerebellar hemorrhage. *Case Report*. A 26-year-old woman presented to the emergency department with sudden headache and hemorrhage of the posterior fossa was diagnosed, probably caused by a vascular malformation. Several hours later, she developed acute pulmonary edema due to acute heart failure. Echocardiography showed left ventricular dysfunction with hypokinetic basal segments and hyperkinetic apex corresponding to inverted takotsubo. Outcome was spontaneously favorable within a few days. *Conclusion*. Inverted takotsubo pattern is a stress-induced cardiomyopathy that could be encountered in patients with subarachnoid hemorrhage and is generally of good prognosis. We described the first case following a cerebellar hematoma.

## 1. Introduction

Stress-induced cardiomyopathy, or takotsubo, is characterized by reversible ventricular dysfunction and mimics acute coronary syndrome with similar symptoms ranging from isolated chest discomfort to, rarely, cardiogenic shock, in the absence of coronary stenosis. The mechanism of takotsubo is still debated, although it seems often triggered by emotional or physical stress. Among these, subarachnoid hemorrhage was previously described as a trigger of importance because heart-related symptoms can precede or be concomitant to cerebral damage and mask neurological symptoms. In the presence of subarachnoid hemorrhage (SAH), takotsubo cardiomyopathy reflects the severity of cerebral hemorrhage and increases the risk of in-hospital death.

To our best knowledge, this is the first report of a case of stress cardiomyopathy presenting with an inverted pattern following a cerebellar hemorrhage.

## 2. Case Description

**Figure 1:**
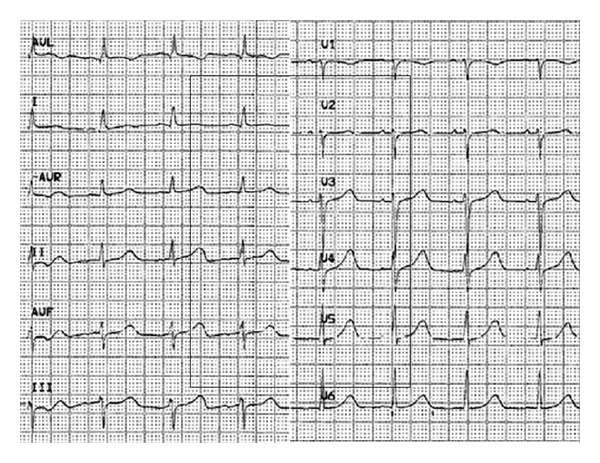
Admission electrocardiogram.

A 26-year-old Caucasian woman without previous medical history was referred to the emergency department for sudden headache. Arterial blood pressure was 110/60 mm Hg and heart rate was 90/min. Because of altered consciousness (Glasgow Coma Scale 6/15), orotracheal intubation was immediately performed. Brain computed tomography (CT) showed an intraparenchymal left cerebellar hemorrhage with massive subarachnoid extension, probably due to a vascular malformation. The first electrocardiogram (ECG) recording in the emergency room showed sinus rhythm with abnormal repolarisation in lateral leads (Figure 1). Troponin-I (Tn-I) values and chest X-ray were normal on admission. The patient underwent immediate surgery for hematoma evacuation and ventricular drainage.

**Figure 2:**
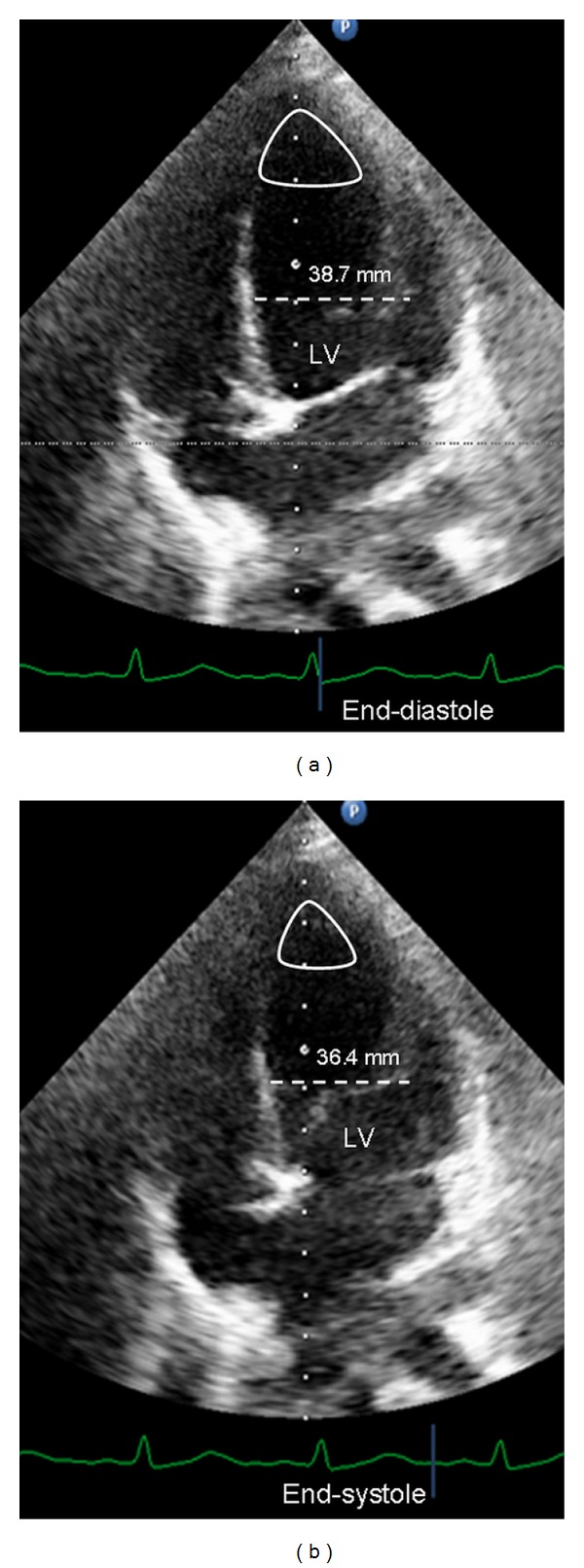
Transthoracic echocardiogram (4-chamber view) during end-diastole (a) and end-systole (b) shows basal and midventricular segmental hypokinesis (dotted line for reduced shortening) of the left ventricle (LV), with a well-preserved apical segmental systolic function (loop).

One day after admission, we observed increasing oxygen requirements and chest X-ray was consistent with pulmonary edema. The ECG at this time was unchanged and serum Tn-I concentration increased till a peak value of 8.25 ng/mL (<0.08 ng/mL). Echocardiography showed normal left ventricular internal dimensions but depressed left ventricular (LV) ejection fraction (25%). LV mid and basal segments were severely hypokinetic, whereas apical segments were hypercontractile (Figure 2 and movie clip 1). The right ventricular size and systolic function, as well as valves, were normal. Nonetheless, the patient did not require hemodynamic support and did not receive catecholamines.

Systolic function steadily improved over the two following days, along with oxygenation parameters and chest X-ray. Extubation was possible on day 3. Echocardiography confirmed the improving LV function. The ECG returned to normal morphology. A control cerebral angiography performed several weeks later failed to reveal the origin of the bleeding. It was hypothesized that a minimal arteriovenous malformation had been removed during the neurosurgical procedure, together with the subsequent hematoma. The patient made a full cardiac and neurological recovery.

## 3. Discussion

Takotsubo cardiomyopathy, also known as transient left ventricular ballooning syndrome or stress-induced cardiomyopathy, is characterized by transient LV dysfunction in the absence of angiographic coronary stenosis and is provoked by an episode of emotional or physical stress. Takotsubo syndrome can mimic acute coronary syndrome presenting with chest pain, T-wave and ST-segment abnormalities on ECG, elevation of troponin level, and left ventricular regional wall motion abnormalities. Stress cardiomyopathy is always reversible.

Four patterns of left ventricles involvement have been described: (1) classical type, (2) reverse type, (3) midventricular type, (4), and localized type. Among them, the classic pattern with LV apical dysfunction is the most frequently encountered by the clinician. Inverted takotsubo was more recently described and is characterized by hypocontractility of basal and midventricular segments. Besides LV echocardiographic patterns, clinical and biological characteristics of inverted takotsubo are quite different than other patterns. Inverted takotsubo commonly presents at an early age and is more often associated with either mental or physical stress [1–3]. Release of troponin is higher compared to other patterns, which is the consequence of the larger muscle region involved in inverse takotsubo compared to apex alone, but, on the other hand, natriuretic peptides is more elevated in apical and midventricular patterns, which is clinically translated by more severe symptoms and higher NYHA functional class [1–3]. Higher prevalence of symptoms despite less cardiomyocytes damage is partially explained by the higher prevalence of mitral regurgitation, associated or not with systolic anterior motion, as the consequence of altered special relationship between mitral leaflets and subvalvular apparatus caused by apical ballooning [4, 5]. Inverted takotsubo also presents with a lower prevalence of T-wave inversion than those with classic patterns.

Among physical stress-triggered cardiomyopathy, SAH is an issue of concern. First of all, takotsubo-related symptoms may be at the forefront and mask neurological symptoms suggesting that echocardiographic findings of regional wall motion abnormalities in multiple coronary artery distributions should make search for cerebral hemorrhage, especially when the patient is a young female [6]. Second, cardiac dysfunction from subarachnoid hemorrhage is a marker of overall poor prognosis as it predominates in severe subarachnoid hemorrhage requiring prolonged intubation and pressor support. Ahmadian et al. showed that onset of myocardial infarction or finding of troponin levels greater than 1 mcg/L increases risk of in-hospital death suggesting that these patients must be very closely monitored and should receive treatment for heart failure with angiotensin converting enzyme inhibitors and beta-blockers [7].

Subarachnoid hemorrhage as a common trigger of apical takotsubo was first described in 2005 by Ennezat et al. in four patients [8]. Since then, 12 cases have been added [6, 8–12]. We described the first case of inverted takotsubo related to a parenchymal hemorrhage, and more specifically to a cerebellar hematoma. This type of cerebral lesion does not* a priori* draw specific attention to heart problem. However, this case illustrates the possible influence of cerebral lesions other than subarachnoid hemorrhage on LV function and should enlarge the field of takotsubo etiologies.

The pathophysiological basis of the myocardial dysfunction is multifactorial. Adrenergic storm is one of the most recognized mechanisms of stress cardiomyopathy, where excessive circulating epinephrine induces multiple coronary spasm, microvascular dysfunction, negative inotropic effect due to anomaly in the intracellular calcium metabolism, and myocardial damage. This hypothesis is reinforced by the fact that catecholamine level is high in the SAH model. The reason of the distribution of myocardial stunning is not yet well understood. Distribution, density, and sensibility of adrenergic receptors seem to play an important role and it was hypothesized that areas with a higher density of adrenergic receptors may determine the area of hypokinesis. This phenomenon explains that typical takotsubo occurs more often in older patients where adrenoreceptors density in the apex is reduced because of hormonal change, while inverted variant occurs in younger patients.

## 4. Limitations

The main limitation of this observation is the absence of coronary angiography, which is requested for the definition of takotsubo. It was not performed for several reasons: (1) the young age of the patient making less likely the presence of a significant coronary artery disease; (2) the rapid clinical, biological, and echocardiographic recovery; (3) the setting of an acute cerebral hemorrhage that could also restrict the use of some medications.

## 5. Conclusion

We describe a case of an inverse takotsubo syndrome after intracerebral bleeding in a young woman. Implication of such a case can be summarized as follows.

- Inverted takotsubo may be the first sign of a neurological disorder, as SAH, but also parenchymal hemorrhage as in our case.
- Despite the young age, ECG, troponin level analysis, and echocardiography should be performed in patients with cerebral hemorrhage and signs of symptomatic left ventricular dysfunction or biological cardiomyocytes necrosis to track stress-induced cardiomyopathy.
- In this setting, acute LV dysfunction has a good outcome with supportive therapy and catecholamines are not indicated.

## Supplementary Material

Transthoracic echocardiogram showing basal and midventricular segmental hypokinesis with preservation of the apical segmental systolic function.
